# Perceived stigma evaluation among residents in psychiatry

**DOI:** 10.1192/j.eurpsy.2024.1662

**Published:** 2024-08-27

**Authors:** R. Masmoudi, S. Ajmi, A. Guermazi, F. Cherif, R. Sallemi, J. Masmoudi

**Affiliations:** Psychiatry A, Hedi Chaker University Hospital, Sfax, Tunisia

## Abstract

**Introduction:**

The nature of psychiatry as a specialty dealing with mental health and emotional well-being may contribute to the perceived stigma. These misconceptions and biases can impact the way psychiatry residents perceive their profession, their own self-esteem, job satisfaction, and overall well-being.

**Objectives:**

Our goal was to evaluate the experience of stigma among psychiatry residents.

**Methods:**

A descriptive cross-sectional online survey was conducted in January 2022 among psychiatry residents at Hedi Chaker University Hospital in Sfax, Tunisia.

The Clinician Associative Stigma Scale (CASS) was used to assess stigmatization experiences.

**Results:**

A total of 34 residents participated in this survey. Their average age was 27.94 years ± 2.43, with 91.2% being female. Of the participants, 61.8% were adult psychiatry residents, and 39.2% were child psychiatry residents. Additionally, the choice of adult psychiatry or child psychiatry specialty was self-determined in 91.2% of cases. The participants had an average of 2 years of experience in psychiatry. They reported a personal medical or surgical history, a personal psychiatric history, and a family history of psychiatric disorders in 32.4%, 8.8%, and 50%, respectively. The average CASS score was 47.09 ± 8.32.

The mean scores for the “discomfort with disclosure” factor, the “stereotypes about mental health professionals” factor, the “negative stereotypes about individuals with serious mental illness” factor, and the “negative stereotypes about effectiveness” factor were respectively 8 ± 3, 9.44 ± 2.57, 15.62 ± 5.7, and 11.35 ± 3.33.

**Conclusions:**

Our study highlighted that residents in psychiatry suffered stigma. Special attention should be given to reducing this phenomenon in this population.

**Disclosure of Interest:**

None Declared

